# Mercury Exposure and Health Impacts among Individuals in the Artisanal and Small-Scale Gold Mining Community: A Comprehensive Review

**DOI:** 10.1289/ehp.1307864

**Published:** 2014-03-28

**Authors:** Herman Gibb, Keri Grace O’Leary

**Affiliations:** Tetra Tech Sciences, Arlington, Virginia, USA

## Abstract

Background: Mercury (Hg) is used in gold mining to extract gold from ore by forming “amalgam”—a mixture composed of approximately equal parts mercury and gold. Approximately 15 million people, including approximately 3 million women and children, participate in artisanal small-scale gold mining (ASGM) in developing countries. Thirty-seven percent of global air emissions of Hg are produced by ASGM. The recently adopted Minamata Convention calls for nations to gather health data, train health-care workers, and raise awareness in regard to ASGM activity.

Objective: The purpose of our review was to evaluate the current literature regarding the health effects of Hg among those working and/or living in or near ASGM communities.

Methods: We searched PubMed, ScienceDirect, and Google Scholar for studies relating to health effects and biomarkers of Hg exposure in ASGM communities. Articles published from 1990 through December 2012 were evaluated for relevance.

Discussion: Studies reporting health assessments, kidney dysfunction, neurological disorders and symptoms, and immunotoxicity/autoimmune dysfunction in individuals living in or near an ASGM community were identified. More than 60 studies that measured biomarkers of Hg exposure in individuals living in or near ASGM communities were also identified. These studies, conducted in 19 different countries in South America, Asia, and Africa, demonstrated that hair and urine concentrations are well above World Health Organization health guidance values in ASGM communities.

Conclusions: ASGM workers and their families are exposed to Hg vapor, and workers, workers’ families, and residents of nearby and downstream communities are consuming fish heavily contaminated with methylmercury.

Citation: Gibb H, O’Leary KG. 2014. Mercury exposure and health impacts among individuals in the artisanal and small-scale gold mining community: a comprehensive review. Environ Health Perspect 122:667–672; http://dx.doi.org/10.1289/ehp.1307864

## Introduction

In February 2009, the Governing Council of the United Nations Environment Programme (UNEP) began development of a legally binding global instrument on mercury (Hg). In January 2013, governments agreed to text for this instrument, thus giving birth to the Minamata Convention on Mercury (UNEP 2013b). In October 2013, the convention was signed in Minamata, Japan. Article 7 and Annex C of the convention address artisanal and small-scale gold mining (ASGM) and the development of national plans for ASGM. Included in the outline for national plans is development of a public health strategy on the exposure of artisanal and small-scale gold miners and their communities to Hg. Such a strategy should include the gathering of health data, training for health-care workers, and raising awareness through health facilities.

Hg is used in gold mining to extract gold from ore by forming “amalgam”—a mixture composed of approximately equal parts Hg and gold [Arctic Monitoring and Assessment Programme (AMAP)/UNEP 2013]. The amalgam is heated, evaporating the Hg from the mixture, leaving the gold (AMAP/UNEP 2013). This method of gold extraction is used in the ASGM community because it is cheaper than most alternative methods, can be used by one person independently, and is quick and easy (UNEP 2012). The dramatic rise in the cost of gold over the last decade has fueled a gold rush by poverty-driven miners in many countries (UNEP 2008). ASGM occurs primarily in South America, Africa, and Asia, but it can also be found in North America and Australia (UNEP 2013c). Approximately 15 million people, including approximately 3 million women and children, participate in the ASGM industry in 70 countries (UNEP 2012). ASGM is the largest source (37%) of global Hg emissions (UNEP 2013c). Between 2005 and 2010, Hg emissions from ASGM doubled (UNEP 2013a). Although most uses of Hg are declining throughout the world, the ASGM demand for Hg is expected to increase (UNEP 2013c). ASGM accounts for the largest percentage of global Hg demand (UNEP 2013c).

Hg vapors in the air around amalgam burning sites can be alarmingly high and almost always exceed the World Health Organization (WHO) limit for public exposure of 1.0 μg/m^3^ (UNEP 2012). These exposures affect not only the workers but also those in the communities surrounding the processing centers (UNEP 2012). Drake et al. (2001) reported that the range of the 8-hr time-weighted average airborne Hg exposure at gold mining operations in Venezuela was 0.1–6,315 μg/m^3^ with a mean of 183 μg/m^3^. The WHO (2000) reported that tremor has been observed in workers exposed to 30 μg/m^3^ Hg and that renal tubular effects and changes in plasma enzymes are estimated to occur at 15 μg/m^3^. The vaporized Hg eventually settles in soil and the sediment of lakes, rivers, bays, and oceans and is transformed by anaerobic organisms into methylmercury (MeHg). In bodies of water, the MeHg is absorbed by phytoplankton, which is ingested by zooplankton and fish, thereby contaminating the food chain. It especially accumulates in long-lived predatory species such as shark and swordfish (WHO 2007).

Elemental Hg and MeHg are toxic to the central and peripheral nervous system. The inhalation of Hg vapor can produce harmful effects on the nervous, digestive, and immune systems and the lungs and kidneys and may be fatal (WHO 2007). Children are especially vulnerable and may be exposed directly by eating MeHg-contaminated fish. MeHg bioaccumulates in fish and when consumed by pregnant women may lead to neurodevelopmental problems in the developing fetus. Transplacental exposure is the most dangerous because the fetal brain is very sensitive (WHO 2007). Neurological symptoms include mental retardation, seizures, vision and hearing loss, delayed development, language disorders, and memory loss. In children, acrodynia, a syndrome characterized by red and painful extremities, has been reported to result from chronic Hg exposure (WHO 2007, 2008).

Elemental Hg, the form of Hg used in gold amalgamation, is a liquid that volatilizes rapidly (WHO 2007). In humans, elemental Hg is typically measured in blood or urine (WHO 2003, 2008). MeHg, the form of Hg that contaminates fish, is typically measured in blood, cord blood, or hair. Sample collection of hair is the preferred method of biomonitoring for MeHg because it is less invasive than blood sampling. Blood Hg concentrations characterize recent or current exposure (WHO 2008). In populations where exposure to Hg occurs primarily through consumption of contaminated fish, most of the total Hg in the blood is organic and can be used as a measure of MeHg exposure (WHO 2008).

Given the prominence of ASGM as a significant source of global Hg and that national plans, as specified by the Minamata Convention, call for the collection of health data, this review was undertaken to identify studies that describe health effects and exposure in ASGM communities. We hoped that such information can serve as a resource to health authorities in those countries where ASGM is currently practiced.

## Methods

To identify relevant studies, we searched PubMed (http://www.ncbi.nlm.nih.gov/pubmed/) as well as ScienceDirect (http://www.sciencedirect.com/) and Google Scholar (http://scholar.google.com/). The following search terms were included: Hg, MeHg, fish consumption, hair, blood, urine, neurological effects, kidney effects, health effects, and gold mining. The names of numerous countries where ASGM is known to occur were also included in searches. A total of 1,317 potentially relevant studies were identified. Studies were considered relevant if they identified health effects or measured hair or urinary Hg concentrations in individuals residing in an area affected by ASGM. We identified 72 studies that fit our criteria for relevance. We divided the studies into two categories: *a*) studies that identified health effects in ASGM communities (Table 1), and *b*) studies that reported hair or urine Hg in ASGM communities or communities affected by ASGM (see Supplemental Material, Tables S1, S2, and S4). Several studies included measurements of blood Hg as well as hair Hg (see Supplemental Material, Tables S2 and S3). We included studies published through December 2012.

**Table 1:** Health effects observed in ASGM areas.

Reference (country of origin)	Study population	Observed effects
Studies that included persons occupationally exposed to Hg from ASGM
Yard et al. 2012 (Peru)	103 people living in a gold mining area, including 35 who had direct contact with Hg at least once per month.	> 50% reported headache, mood swings, or muscle weakness. Previous medical diagnoses included digestive system disorder (*n* = 20), kidney dysfunction (*n* = 9), and nervous system disorders (*n* = 4). Participants reporting kidney dysfunction had higher urine total Hg concentrations (GM = 12.0 μg/g-creatinine) than those not reporting kidney dysfunction (GM = 5.1 μg/g-creatinine; *p *< 0.05). Urinary Hg concentrations were significantly (*p* < 0.05) higher among people who heated amalgam compared with those who never heated amalgam.
Harari et al. 2012 (Ecuador)	200 gold miners; 37 gold merchants; 72 referents.	Tremor was found to be associated with blood and urinary Hg. Elimination of Hg appeared to be modified by a polymorphism. The gold merchants had the highest blood and urinary Hg because they burned amalgam on a daily basis.
Tomicic et al. 2011 (Burkina Faso)	93 gold workers; 779 workers related to gold mining activities.	Nearly half of the 93 workers considered most susceptible to Hg exposure reported ≥ 5 symptoms possibly related to Hg exposure (frequent headaches, sleep disorder, dizziness/fits of giddiness, wounds/irritation of mouth, unusual tiredness, walking difficulty, trembling, pins and needles/tingling in hands or feet, vision disorder, persistent cough, thoracic pain, rhinitis). Gold ore dealers were found to have higher urine Hg than ore washers; dealers heated Hg an average of 13.2 times per day compared with individuals not dealing gold who heated Hg 7.8 times per day.
Bose-O’Reilly et al. 2010a (Indonesia)	Group 1: 21 Sulawesi residents (control group); Group 2: 66 Kalimantan residents living in an exposed area with no occupational exposure; Group 3: 18 Sulawesi residents living in an exposed area with no occupational exposure; Group 4: 30 Kalimantan Hg workers—panning, but no smelting; Group 5: 17 Sulawesi, Hg workers—panning, but no smelting; Group 6: 69 Kalimantan Hg workers—smelting; Group 7: 60 Sulawesi, Hg worker–smelting.	A determination of Hg intoxication was based on a merging of medical score and biomonitoring results. The following rates of intoxication were observed: Group 1: 0%; Group 2: 31:8%; Group 3: 16.7%; Group 4: 43.3%; Group 5: 23.5%; Group 6: 62.3%; Group 7: 41.6%. Hg-exposed workers showed typical symptoms of Hg intoxication, such as movement disorders (ataxia, tremor, dysdiadochokinesia, etc.).
Gardner et al. 2010 (Brazil)	98 gold miners in Rio Rato (Para state); 91 emerald miners (Goias state); 57 diamond miners (Goias state).	Hg-exposed gold miners had higher prevalence of detectable ANA and ANoA as compared with diamond and emerald miners with no occupational Hg exposure. Hg-exposed gold miners with detectable ANA or ANoA in serum had significantly higher serum concentrations of the proinflammatory cytokines IL-1β, TNF-α, and IFN-γ as compared with diamond and emerald miners. The authors concluded that the results suggest that Hg increases autoimmune dysfunction and systemic inflammation.
Bose-O’Reilly et al. 2008 (Indonesia, Zimbabwe)	80 children working with Hg (51 Indonesia, 29 Zimbabwe); 36 children living in Hg-exposed areas (22 Indonesia, 14 Zimbabwe); 50 [control group (31 Indonesia, 19 Zimbabwe)].	8% of children working with Hg in Indonesia were considered Hg intoxicated. 29% of children living in Hg-exposed areas in Zimbabwe were considered intoxicated. 55% of children working with Hg in Zimbabwe were considered intoxicated. None of the control children were considered intoxicated. Chronic Hg intoxication is a combination of severe and specific symptoms and raised Hg levels in biomarkers.
Silva et al. 2004; Brazil)	98 (54 currently working in a gold mine) in Rio Rato, a gold mining community in the mid-Tapajos watershed; 140 in Jacareacanga, a riverine settlement on the mid-Tapajos River (no current occupational exposure; elevated MeHg in fish consumed); 98 in Tabatinga, a riverine community in the lower Amazon (no occupational exposure; low MeHg in fish).	There was a high prevalence of detectable ANA (54.1%) and ANoA (40.8%) in Rio Rato miners (≥ 1:10 serum dilution). The prevalence was lower in Jacareancanga (10.7% ANA and 18% ANoA) and much lower in Tabatinga (7.1% ANA and 2.0% ANoA).
Drake et al. 2001 (Venezuela)	21 small-scale gold miners.	3 had detectable NAG, a biological marker of preclinical, nonspecific damage to the kidney’s proximal tubule cells in excess of reference values.
Drasch et al. 2001 (Philippines)	102 occupationally exposed ball-millers and amalgam-smelters; 63 Mt. Diwata residents, environmentally exposed; 100 individuals residing downstream in Monkayo; 42 Davao residents (control group).	Workers reported fatigue, tremor, memory problems, restlessness, weight loss, metallic taste, and disturbances in sleeping. Diagnosis of Hg intoxication in the workers was significantly higher in the downstream community, in the Mt. Diwata nonoccupational group, and in the workers compared with the control group. Median concentrations of Hg in blood, urine, and hair among the workers were significantly different from the concentrations found in the control group.
Studies of persons not engaged in gold mining but living in areas affected by ASGM
Nyland et al. 2011 (Brazil)	232 persons living in the Lower Tapajós River Basin in the Brazilian Amazon, an area with a history of Hg use in small-scale gold mining.	Elevated titers of Hg in blood and plasma were associated with ANA but not with ANoA. Pro-inflammatory and antinflammatory interleukins and IL-17 were increased with MeHg exposure but were decreased in the subset of the population with elevated ANA. The authors concluded that the data indicate an immunotoxic or immunomodulatory effect of MeHg in the range of exposures found in this population but suggest that there may be a specific phenotype of MeHg susceptibility.
Tian et al. 2009 (China)	203 persons living in an area contaminated with Hg due to gold extraction; 191 persons from a control area.	Urinary Hg was significantly correlated with β_2_-microglobulin and NAG.
Alves et al. 2006 (Brazil)	105 adults in seven riverine communities (104 consumed fish daily); six riverine communities were along the mid-Rio Negro; one was located along the tributary Rio Cuiuni; 105 controls (volunteer donors at a blood bank); fish species high in MeHg were consumed more frequently by the riverine population (45.5%) than by controls (18.8%).	Mean hair Hg of the riverines (34.5 ppm) was significantly higher than controls (1.0 ppm). Positive serum ANA was more frequently observed in riverine fish-eaters (12.4%) than controls (2.9%). There was, however, no significant association between hair Hg and ANA.
Cordier et al. 2002 (French Guiana)	156 children and their 104 mothers in Upper Maroni communities (high Hg exposure); 69 children and their 51 mothers in Camponi (medium Hg exposure); 153 children and their 115 mothers in Awala (low Hg exposure). (Hg exposure was the result of gold mining activity.)	No major neurological signs were observed in the children examined. After adjusting for potential confounders, a dose-dependent association was observed between maternal hair Hg level and increased deep tendon reflexes, poorer coordination of legs, and decreased performance in the Standard-Binet Copying score, which measures visuospatial organization.
Harada et al. 2001 (Brazil)	132 fishermen and their families (*n* = 182) in three fishing villages along the Tapajos River.	General sensory disturbance was observed in 16 of 50 subjects (32%) whose hair Hg was > 20 ppm. Several subjects were diagnosed with Minamata disease.
Akagi et al. 2000 (Philippines)	162 school­children in a community of Mindanao where gold is processed.	Predominant findings among the children were gingival discoloration, adenopathy, underweight, and dermatologic abnormalities.
Grandjean et al. 1999 (Brazil)	351 school­children between 7 and 12 years of age in four villages along the Tapajos River basin downstream from a gold mining area; 135 mothers of 252 children were examined; hair samples obtained from 113 mothers of 222 children were examined.	Neuropsychological tests of motor function, attention, and visuospatial performance showed decrements with increasing hair Hg concentrations.
Lebel et al. 1998 (Brazil)	91 adult inhabitants living 250 km downstream from the most extensive gold mining fields in Brazil. The villagers were not exposed to Hg vapor. The area was accessible only by water.	Near visual contrast sensitivity and manual dexterity, adjusted for age, decreased significantly with increasing hair Hg levels. There was a tendency for muscular fatigue to increase and muscular strength to decrease in women. Hair Hg levels were significantly higher for persons who presented disorganized movements on an alternating movement task and for persons with restricted visual fields. Hair Hg concentrations were < 50 μg/g.
Abbreviations: ANA, antinuclear autoantibodies; ANoA, anti-nucleolar autoantibodies; GM, geometric mean; IFN interferon; IL, interleukin; NAG, *N*-acetyl-β-d-glucoaminidase; TNF, tumor necrosis factor.		

We compared hair concentrations with 2.5 μg/g, the hair concentration associated with the provisional tolerable weekly intake (PTWI) [Bellanger et al. 2013; Food and Agricultural Organization of the United Nations (FAO)/WHO 2003]. The PTWI was established to protect the developing fetus from neurotoxic effects (WHO 2007). In 2006, the Joint FAO/WHO Expert Committee on Food Additives (JECFA) concluded that life stages other than the embryo and fetus may be less sensitive to the effects of MeHg. Available data did not allow the JECFA to make firm conclusions with respect to children ≤ 17 years of age and therefore stated that the PTWI also applied to children (WHO 2007).

In the present study, we compared urine concentrations with 50 μg Hg/g-creatinine, a concentration at which renal tubular effects and changes in plasma enzymes are expected to occur (WHO 2000). Urine concentrations were also compared with 100 μg Hg/g-creatinine, a urinary concentration at which the probability of developing the classical neurological signs of Hg intoxication is high (WHO 1991). For purposes of presentation, only studies that reported ranges and mean urinary or hair Hg were included in figures to demonstrate the extremes of Hg exposure. All study results, however, are presented in Supplemental Material, Tables S1–S4.

## Results

We identified 17 studies in the literature that described health effects in ASGM communities (Table 1). The health effects studies were conducted in 10 different countries on three continents (South America, Africa, and Asia). All of the studies were cross-sectional.

Numerous biomarker (hair, blood, urine) studies have been conducted in ASGM populations. The biomarker studies were conducted in 19 different countries on three continents. Studies that reported hair Hg concentrations among residents of ASGM communities, miners, and environmentally exposed populations are reported in Supplemental Material, Table S1. These studies were conducted in 14 different countries. Supplemental Material, Table S2, includes studies that examined hair and blood Hg in the same population; 8 different countries are represented by these studies. Supplemental Material, Tables S3 and S4 contain studies that reported Hg concentrations in blood and urine, respectively. The studies on blood and urinary Hg were conducted in 5 and 13 countries, respectively.

**Figure 1:**
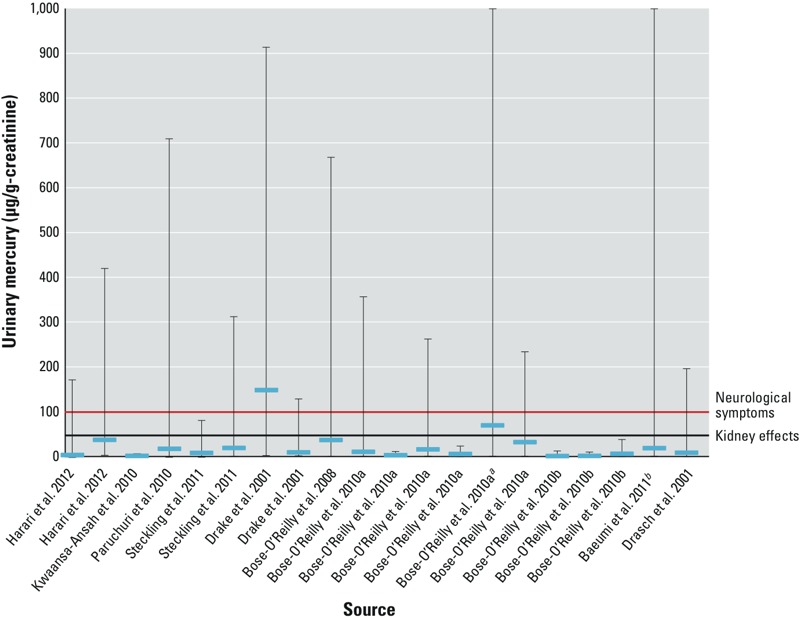
Means (horizontal blue lines) and ranges (vertical lines) of urinary Hg found in ASGM populations. The red line indicating “Neurological symptoms” designates the 100 μg/g-creatinine value for neurological effects identified by WHO (1991). The black line indicating “Kidney effects” designates the 50 μg/g-creatinine concentration at which renal tubular effects are expected to occur (WHO 2000). For more information, see Supplemental Material, Table S4. References that appear on the *x*-axis more than once indicate that the study included more than one study group [e.g., merchants and miners in Harari et al. (2012)]. ***^a^***The highest value for data in this category was 1697.39 μ/g-creatinine. ***^b^***The lowest value for data in this study was < LOD; the highest value was 1697 μg/g-creatinine.

Figure 1 shows urinary Hg concentrations from studies where both a mean (horizontal blue line) and a range (vertical line) were reported. “Neurological symptoms” designates the 100 μg/g-creatinine value for neurological effects identified by WHO (1991). “Kidney effects” designates the 50 μg/g-creatinine concentration at which renal tubular effects are expected to occur (WHO 2000).

**Figure 2:**
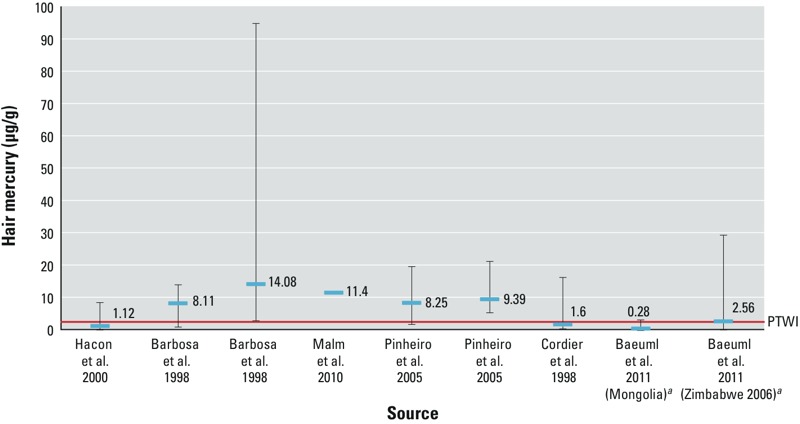
Means (horizontal blue lines) and ranges (vertical lines) of hair Hg of women in studies of residents of ASGM communities. PTWI, provisional tolerable weekly intake. For more information, see Supplemental Material, Table S1. ***^a^***The lowest value for these studies was < LOD.

**Figure 3:**
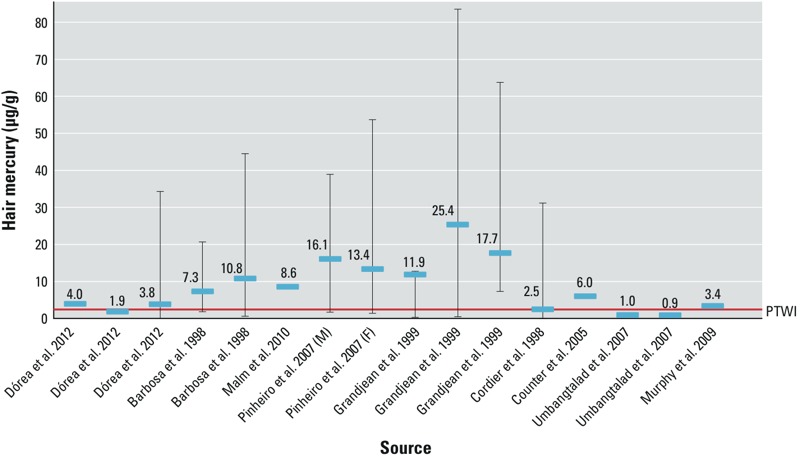
Means (horizontal blue lines) and ranges (vertical lines) of hair Hg of children and infants in studies of residents of ASGM communities. PTWI, provisional tolerable weekly intake. For more information, see Supplemental Material, Table S2.

Figure 2 shows hair Hg concentrations in female residents of ASGM communities from studies where both a mean and a range (where shown) were reported. Most of the means and all of the maximum concentrations reported were above the PTWI. Figure 3 shows means and ranges of hair Hg of children and infants in ASGM communities. Women and children were selected for Figures 2 and 3, respectively, because the developing fetus and children are considered more vulnerable to the effects of MeHg.

## Discussion

The most common health effect reported among workers engaged in ASGM are neurological effects (Table 1). These include tremor, ataxia, memory problems, and vision disorder and were found to occur not just among those engaged in mining activities but also among fish consumers living downstream of mining activities.

Increased urinary excretion of the enzyme *N*-acetyl-β-d-glucoaminidase (NAG), a biomarker of damage to the proximal tubules of the kidney, was found among occupationally exposed individuals (Drake et al. 2001) as well as among those living in a community where gold mining had been practiced (Tian et al. 2009). Kidney dysfunction was clinically diagnosed in 9 of 103 individuals in a gold mining population in Peru (Yard et al. 2012). Those reporting kidney dysfunction had higher urine total Hg concentrations [geometric mean (GM) = 12.0 μg/g-creatinine] than those not reporting kidney dysfunction (GM = 5.1 μg/g-creatinine; *p* < 0.05). See Table 1 for further information on kidney effects.

Four studies conducted in Brazil suggest that Hg exposure among gold miners and in gold mining communities is associated with an increase in the prevalence of markers of autoimmune dysfunction (Alves et al. 2006; Gardner et al. 2010; Nyland et al. 2011; Silva et al. 2004). See Table 1 for the details of each study.

Ten of the study populations in Table 1 were in South America, six were in Brazil alone. Only two of the study populations are in Africa; four are in Asia. The number of studies in a geographic region should not be construed to represent the magnitude of the problem in that region, however. The number of artisanal and small-scale gold miners and the amount of Hg released from ASGM is as great, if not greater, a problem in Asia and Africa as it is in South America (UNEP 2013c).

Harari et al. (2012), Steckling et al. (2011), Tomicic et al. (2011), Paruchuri et al. (2010), Bose-O’Reilly et al. (2008, 2010a), Counter et al. (2006), and Drake et al. (2001) all reported urinary Hg concentrations well above 100 μg Hg/g-creatinine. Data on urinary concentrations of Hg are provided in Figure 1 and Supplemental Material, Table S4. The WHO (1991) considers 100 μg Hg/g-creatinine to be the level above which the probability of developing classical neurological signs of Hg intoxication is high. High urinary Hg concentrations were particularly evident among those who amalgamate Hg or heat Hg to remove it from the amalgam. As an example of the elevated urinary concentrations found among small-scale gold mining operations, Tomicic et al. (2011) reported that the mean urinary Hg among gold dealers in Burkina Faso was 299.1 μg Hg/g-creatinine. Gold dealers were believed to have the most frequent exposure to Hg vapor. Drake et al. (2001) reported that among self-employed gold miners in Venezuela, the mean urinary Hg concentration was 148 μg Hg/g-creatinine; the high end of the range was 912 μg Hg/g-creatinine. Bose-O’Reilly et al. (2008) reported that the mean urinary Hg concentration among a sample of 80 children working with Hg was 36.50 μg Hg/g-creatinine; the high end of the range was 666.87 μg Hg/g-creatinine. The children who worked in small-scale gold mining operations in Indonesia and Zimbabwe ranged in age from 9 to 17 years. Umbangtalad et al. (2007) found that Thai schoolchildren living near, but not working in, small-scale gold mining operations had increased urinary Hg concentrations.

Hair Hg reflects the ingestion of Hg from fish (MeHg) (WHO 1990). The mean hair Hg concentration in virtually all of the 55 studies conducted in ASGM areas or areas affected by ASGM are above the concentration (2.5 μg/g) associated with the WHO’s PTWI (Figures 2 and 3; see also Supplemental Material, Tables S1 and S2). Many of the studies reported hair concentrations > 14 μg/g, which the FAO/WHO (2003) considers a no observed effects level (NOEL). The NOEL is based on neurotoxic effects in the fetus; the PTWI was established to protect the fetus from neurotoxic effects (WHO 2007). In 2006, the JECFA concluded that life stages other than the embryo and fetus may be less sensitive to the effects of MeHg. Because of a lack of data on children < 17 years of age, the JECFA concluded that the PTWI should also apply to children (WHO 2007). Hair Hg concentrations above those that reflect the PTWI and NOEL should, therefore, not be indicative of health effects in adults. The WHO (1990) concluded that a hair Hg concentration of 50 μg Hg/g would indicate a low (5%) risk of neurological damage to adults. Some of the studies listed in Table 1 [e.g., Harada et al. (2001) and Lebel et al. (1998)], however, suggest that neurological effects may be evident in adults at hair Hg concentrations of < 50 μg Hg/g. Blood concentrations of Hg may reflect exposure to either MeHg or inorganic Hg (WHO 1990, 2003). The background level of Hg in blood for those who do not eat fish is 2 μg Hg/L (WHO 2003).

## Conclusions

Individuals involved in the gold mining operations, their families, and those in the gold mining communities are exposed to dangerous levels of elemental Hg vapor, as evidenced by urinary Hg concentrations. This evidence includes extremely elevated urinary Hg concentrations in children who work in the mines and children who live in the areas where small-scale gold mining occurs. Residents in the gold mining communities and downstream of the gold mining communities consume fish that may be heavily contaminated with MeHg, as demonstrated by hair Hg measurements. Current studies indicate that those in the ASGM communities experience neurological effects, kidney effects, and possibly immunotoxic/autoimmune effects from Hg exposure. Not only is the danger widespread globally, but the problem is expected to grow. National public health strategies on ASGM, as required by the Minamata Convention, should be implemented immediately.

## Supplemental Material

(433 KB) PDF
