# Supplementary material for: Mercury Exposure and Health Impacts among Individuals in the Artisanal and Small-Scale Gold Mining Community: A Comprehensive Review
Source: Environ Health Perspect. 2014 Mar 28;122(7):667–72. doi: 10.1289/ehp.1307864 (PMC4080518; doi:10.1289/ehp.1307864)
Supplement: (433 KB) PDF [file ehp.1307864.s001.pdf]

Environmental Health Perspectives doi:10.1289/ehp.1307864

Supplemental Material

Mercury Exposure and Health Impacts among Individuals in the Artisanal and Small-Scale Gold Mining Community: A Comprehensive Review

Herman Gibb and Keri Grace O'Leary

Table of Contents **Page**

Table S1. Studies that Reported Hair (µg/g) Mercury Among Residents of ASGM Communities, Miners, and Environmentally Exposed Populations **2**

Table S2. Studies that Reported Hair (µg/g) & Blood (µg/L) Mercury Concentrations Among Residents of ASGM Communities, Miners, and Environmentally Exposed Populations **9**

Table S3. Studies that Reported Blood Mercury Concentrations (µg/L) Among Residents of ASGM Communities, Miners, and Environmentally Exposed Populations **14**

Table S4. Studies that Reported Urinary Mercury Concentrations (µg/g-Cr unless otherwise specified) Among Residents of ASGM Communities and Miners **15**

Table S1. Studies that Reported Hair (μg/g) Mercury Among Residents of ASGM Communities, Miners, and Environmentally Exposed Populations.

Source	Location	N	Geometric Mean Hg ± SD (Range)[a]	Mining/Fish Consumers	Population Notes
Barbieri et al. 2009	Bolivia	150	3.02 (0.42-15.65)	Y/Y	Sample of residents of intense gold mining community
Maurice-Bourgoin et al. 2000	Bolivia	80	9.81 (4.30-19.52)	Y/Y	Indigenous communities on banks of Beni River
Maurice-Bourgoin et al. 2000	Bolivia	80	0.28 (0.02-1.02)	Y/Y	Gold miners from the K'aka River
Monrroy et al. 2008	Bolivia	556	5.3±4.3 (0.08-34.1)	N/Y	Amerindian populations living along Beni River
Dorea et al. 2012	Brazil	33	3.95±1.8	N/Y	Itapuã; infants from former subsistence fisherman families
Dorea et al. 2012	Brazil	166	1.85±0.9	Y/Y	Bom Futuro; infants from families of cassiterite mining families
Dorea et al. 2012	Brazil	82	3.84±5.5	N/Y	Porto Velho; infants from urban families
Hacon et al. 2000	Brazil	75[b]	1.12±1.17 (0.05-8.20)	Y/N	Alta Floresta, Mato Grosso state (gold mining area); pregnant women who were patients at the prenatal Health Community Center
Barbosa et al. 1998	Brazil	28[b]	8.11±3.16 (0.8-13.70)	Y/Y	Indigenous women; Indians (Kayapó reservation, Fresco River)
Barbosa et al. 1998	Brazil	98[b]	14.08±12.80 (2.6-94.70)	Y/Y	Indigenous women; non-Indians (Madeira River)
Barbosa et al. 1998	Brazil	54	7.30±3.50 (2.0-20.40)	Y/Y	Indigenous children; Indians
Barbosa et al. 1998	Brazil	71	10.82±8.46 (0.8-44.4)	Y/Y	Indigenous children; non-Indians
Santos et al. 2002	Brazil	324	16.01±18.92 (4.50-90.40)	Y/Y	Sai Cinza, State of Para; Munduruku Indians
Malm et al. 1995	Brazil	10	25 (5.7-52)	Y/Y	Jacareacanga – on Tapajós River Basin, mercury-contaminated
Malm et al. 1995	Brazil	55	26 (4.7-151)	Y/Y	Brasília Legal – located 250 km downstream from gold mining
Malm et al. 1995	Brazil	46	12	Y/Y	Ponta de Pedra – small fishing village

Source	Location	N	Geometric Mean Hg ± SD (Range)[a]	Mining/Fish Consumers	Population Notes
Malm et al. 1995	Brazil	10	2.7	Y/Y	Santarém – 800 km downstream from gold mining
Guimarães et al. 1999	Brazil	15	28	Y/Y	Amapá, Duas Bocas Lake; fishermen and families
Guimarães et al. 1999	Brazil	15	16.7	N/Y	Amapá, Pracuúba Lake; fishermen and families
Malm et al. 2010	Brazil	32[b]	THg: 15.8±8.3<br>MeHg: 11.4±4.7	Y/Y	Barreiras, Tapajós river basin mothers; 4 cm hair segment (corresponding with months 0-4 of study)
Malm et al. 2010	Brazil	49	THg: 11.4±4.6<br>MeHg: 8.6±3.5	Y/Y	Barreiras, Tapajós river basin, children (of mothers in the study); 4 cm hair segment (0-4 months)
Dórea et al. 2005a	Brazil	77	2.87±2.13	Y/N	Munduruku tribe members residing in Kuburua village
Dórea et al. 2005a	Brazil	86	4.76±2.09	Y/N	Munduruku tribe members residing in Cururu village
Dórea et al. 2005a	Brazil	40	16.55±11.44	Y/Y	Kayabi tribe members
Dórea et al. 2005b	Brazil	89	2.5±1.4	Y/N	Eastern Amazon indigenous tribe (Munduruku) located on river banks, Kaburua village residents
Dórea et al. 2005b	Brazil	138	3.7±1.6	Y/N	Cururu village residents
Dórea et al. 2005b	Brazil	22	6.0±2.9	Y/N	Terra Preta village residents
Dórea et al. 2005b	Brazil	47	12.8±7.0	Y/Y	Eastern Amazon indigenous tribe (Kayabi) located on river banks, Kayabi village residents
Pinheiro et al. 2005	Brazil	19[b]	8.25 (1.51-19.43)	Y/Y	Riverside communities of the Tapajós river basin; pregnant women
Pinheiro et al. 2005	Brazil	21[b]	9.39 (5.25-21.00)	Y/Y	Riverside communities of the Tapajós river basin; non-pregnant women
Pinheiro et al. 2007	Brazil	48	Males: 16.07±8.48 (1.99-38.80);<br>Females: 13.39±9.08 (1.34-53.80)	Y/Y	São Luis do Tapajós residents; Tapajós river basin; mercury exposed region (children aged 1 mo. to 10 years)
Pinheiro et al. 2007	Brazil	84	Males: 9.13±4.71 (1.66-17.70);<br>Females: 6.48±2.61 (1.33-12.10)	Y/Y	Barreiras, Tapajós river basin, residents; mercury exposed region (children aged 2 mo. to 11 years)
Pinheiro et al. 2007	Brazil	36	Males: 2.23±1.57 (0.39-5.16);<br>Females: 2.74±0.96 (0.85-9.46)	N/Y	Panacauera, Tocantins river basin, residents; non-exposed region

Source	Location	N	Geometric Mean Hg ± SD (Range)[a]	Mining/Fish Consumers	Population Notes
Harada et al. 2001	Brazil	76	16.4±10.6 (1.8-53.8)	Y/Y	Barreiras – Tapajós river fishing village residents
Harada et al. 2001	Brazil	12	14.1±9.3 (3.1-34.5)	Y/Y	Rainha - Tapajós river fishing village residents
Harada et al. 2001	Brazil	44	20.8±10.6 (5.1-42.2)	Y/Y	São Luis do Tapajós - Tapajós river fishing village residents
Leino & Lodenius 1995	Brazil	125	35 (0.9-240)	Y/Y	Exposed and referent groups combined; near Tucuruí water reservoir
Pinheiro et al. 2006	Brazil	32	14.8±2.2	Y/Y	São Luis do Tapajós residents
Pinheiro et al. 2006	Brazil	37	15.1±2.8	Y/Y	Barreiras, Tapajós river basin, residents
Pinheiro et al. 2006	Brazil	22	3.0±0.3	N/Y	Panacauera residents
Pinheiro et al. 2006	Brazil	43	7.1±2.1	N/Y	Pindobal Grande residents
Crompton et al. 2002	Brazil	205	Males: 11.0; Females: 6.7	Y/Y	Jacareacanga residents
Grandjean et al. 1999	Brazil	105	3.8 (0.5-12.4)	N/Y	Comparison/referent – Santana do Ituqui children
Grandjean et al. 1999	Brazil	91	11.9 (0.7-35.8)	Y/Y	Brasilia Legal children (less exposed)
Grandjean et al. 1999	Brazil	87	25.4 (0.6-83.5)	Y/Y	São Luis do Tapajós children (more exposed)
Grandjean et al. 1999	Brazil	71	17.7 (7.3-63.8)	Y/Y	Sai-Cinza children (more exposed)
Barbosa et al. 1997	Brazil	55	34.2 (93% above 10)	Y/Y	Apiacas Reserve and surrounding area residents
Lebel et al. 1997	Brazil	96	Males: 15.7 (median); Females: 11.2 (median)	Y/Y	Brasilia Legal residents, many fishermen; downstream from gold mining
Olivero-Verbel et al. 2011	Colombia	1328	1.56±0.06 (0.01-20.14)	Y/Y	Cauca and Magdalena rivers; various distances from the Bolivar gold mining area
Peplow & Augustine 2012	Suriname	158	14.0±6.0	Y/Y	Puleowime (Apetina) residents
Peplow & Augustine 2012	Suriname	106	9.0±4.0	Y/Y	Kawemhakan (Anapayke) residents
Fujimara et al. 2012	French Guiana	387	Males: 9.4 (2.3-26.4); Females: 9.9 (1.6-26.6)	Y/Y	Upper Maroni; Amerindians
Fujimara et al. 2012	French Guiana	77	2.6	N/Y	Referent group; Atlantic coast
Cordier et al. 2002	French Guiana	104	12.7	Y/Y	Upper Maroni (high exposure)
Cordier et al. 2002	French Guiana	115	2.8	Y/Y	Awala (medium exposure)

Source	Location	N	Geometric Mean Hg ± SD (Range)[a]	Mining/Fish Consumers	Population Notes
Cordier et al. 2002	French Guiana	51	6.7	Y/Y	Camopi (low exposure)
Cordier et al. 1998	French Guiana	255	3.4 (0.2-22.0)	Y/Y	Adult residents selected from maternity hospitals and health centers
Cordier et al. 1998	French Guiana	109[b]	1.6 (0.2-16.0)	Y/Y	Pregnant residents
Cordier et al. 1998	French Guiana	136	2.5 (0.2-31.0)	Y/Y	Child residents
Fréry et al. 2001	French Guiana	165	11.4±4.3 (maximum: 27.20)	Y/Y	Wayana Amerindian population living along the upper Maroni River
Ashe 2012	Peru	104	Males: 3.39; Females: 2.23	Y/Y	Puerto Maldonado (less likely to be involved in mining)
Ashe 2012	Peru	100	Males: 2.30; Females: 1.37	Y/Y	Sample of residents from mining regions in Madre de Dios
Counter et al. 2005	Ecuador	80	6.0	Y/N	Nambija; Andean children of Saraguro and Metizo gold miners
Lasut et al. 2010	Indonesia	28	2.9±0.7 (0.5-7.8)	Y/Y	Buyat Pante village residents; submarine tailings disposal site for industrial gold mine
Lasut et al. 2010	Indonesia	29	1.0±0.3 (0.4-1.5)	N/Y	Bajo Village in North Sulawesi, reference group
Sakamoto 2004	Indonesia	35	THg: 2.65 (0.77-7.64); MeHg: 2.42 (0.53-7.73)	Y/Y	Villagers living nearby Buyat Bay; tailings are being deposited into the bay
Sakamoto 2004	Indonesia	16	THg: 3.72 (1.54-9.65); MeHg 3.22 (0.61-9.60)	Y/Y	Villagers living nearby Totok Bay; reference group
Umbangtalad et al. 2007	Thailand		1.07±0.5 (SE)	N/N	Individuals residing in non-gold mining areas with no exposure; control group
Umbangtalad et al. 2007	Thailand	17	1.22±0.04	Y/N	Phanom Pha; miners who worked in the amalgamation process
Umbangtalad et al. 2007	Thailand	62	1.16±0.01	Y/N	Phanom Pha; miners who worked in the ore preparation area
Umbangtalad et al. 2007	Thailand	40	0.95±0.02	Y/N	Phanom Pha; elementary school students – involved directly or indirectly in gold mining activities
Umbangtalad et al. 2007	Thailand	19	0.90±0.03	Y/N	Phanom Pha; elementary school students not involved or associated with mining

Source	Location	N	Geometric Mean Hg ± SD (Range)[a]	Mining/Fish Consumers	Population Notes
Murphy et al. 2008	Cambodia	59	4.01±0.36	N/Y	Mekong River residents; adults
Murphy et al. 2008	Cambodia	19	3.38±0.27	N/Y	Mekong River residents; children
Murphy et al. 2008	Cambodia	3	2.93±1.1	Y/N	O Tron mine workers
Murphy et al. 2008	Cambodia	13	2.33±0.43	Y/N	Prey Meas mine workers
Murphy et al. 2008	Cambodia	9	5.02±1.34	Y/N	Phnom Penh goldsmiths
Saeki et al. 1996	Papua New Guinea	31	0.55 (0.19-1.10)	N/N	Wau-Bulolo area, Morube province; Manki compound residents
Saeki et al. 1996	Papua New Guinea	49	0.55 (0.25-1.10)	N/N	Wau-Bulolo area, Morube province; Wandumi and Kaisenik compunds (upstream from mining)
Saeki et al. 1996	Papua New Guinea	35	0.84 (0.39-1.90)	Y/N	Misbut and Kiroro compounds (part-time gold mining workers)
Saeki et al. 1996	Papua New Guinea	23	0.9 (0.49-2.00)	Y/N	Sambio compound (part-time gold mining workers)
Saeki et al. 1996	Papua New Guinea	24	1.8 (0.93-3.00)	Y/N	Namie and Kapin compounds (mostly full-time mining workers)
Saeki et al. 1996	Papua New Guinea	22	1.4 (0.68-2.2)	Y/N	Chiatz compound (downstream from mining activities but not involved)
Williams et al. 2000	Philippines	48	Males: 2.8 (0.31-13.00)	Y/N	Apokon, Mindanao residents; small-scale gold mining area
Williams et al. 2000	Philippines	130	3.7 (<0.1-148.5)	Y/N	Honda Bay area, Palawan residents
Donkor et al. 2006	Ghana	123	1.11±0.63 (0.39-4.13)	Y/Y	Pra River Basin; volunteers from environmental testing sites, mostly miners (Rainy Season)
Donkor et al. 2006	Ghana	7	3.20±2.03 (0.89-6.50)	Y/Y	Pra River Basin; volunteers from environmental testing sites, mostly miners (Dry Season)
Kwaansa-Ansah et al. 2010	Ghana	40	2.14±1.53 (0.57-6.07)	Y/Y	Dunkwa-On-Offin; miners
Kwaansa-Ansah et al. 2010	Ghana	54	2.35±1.58 (0.63-7.19)	Y/Y	Sample of farmers

Source	Location	N	Geometric Mean Hg ± SD (Range)[a]	Mining/Fish Consumers	Population Notes
Paruchuri et al. 2010	Ghana	120	1.1±3.2 (0.0-22.29)	Y/N	Talensi-Nabdam district, Upper East region; miners and residents
van Straaten 2000	Tanzania	36	0.1-0.6	Y/Y	Sample of populations around small scale gold mining camps (Northern Tanzania) and the southern shores of Lake Victoria
van Straaten 2000	Tanzania	2	18.5-288	N/Y	Control group: people from outside the mining area; selected samples (THg levels most likely from banned skin and hair cosmetics)
Harada et al. 1999	Tanzania	21	81.9±241 (0.38-953)	Y/Y	Employees at gold mines around Lake Victoria; Imwelv
Harada et al. 1999	Tanzania	18	3.35±3.47 (0.38-9.7)	Y/Y	Imwelv (outliers excluded)
Harada et al. 1999	Tanzania	17	1.31±1.58 (0.33-7.0)	Y/Y	Chipaka
Harada et al. 1999	Tanzania	7	1.01±0.52 (0.29-1.8)	Y/Y	Mutakuja
Harada et al. 1999	Tanzania	22	5.42±10.8 (0.61-51.0)	Y/Y	Katoma
Harada et al. 1999	Tanzania	21	3.29±3.64 (0.61-14.3)	Y/Y	Katoma (outliers excluded)
Harada et al. 1999	Tanzania	14	3.62±5.52 (0.83-22.0)	Y/Y	Buckleef
Harada et al. 1999	Tanzania	15	2.00±2.08 (0.73-9.5)	Y/Y	Ikungu
Harada et al. 1999	Tanzania	18	9.00±21.6 (0.57-94.3)	Y/Y	Simbasirori
Harada et al. 1999	Tanzania	17	4.03±4.12 (0.57-13.5)	Y/Y	Simbasirori (outliers excluded)
Harada et al. 1999	Tanzania	18	1.02±1.12 (0.31-5.1)	Y/Y	Employees at a gold mine (Dodoma) inland a long distance from Lake Victoria (first visit)
Harada et al. 1999	Tanzania	18	12.1±44.4 (0.28-190)	Y/Y	Dodoma (second visit)
Harada et al. 1999	Tanzania	17	1.61±2.03 (0.28-8.6)	Y/Y	Dodoma (second visit-outliers excluded)
Harada et al. 1999	Tanzania	15	8.23±19.6 (0.40-70.4)	Y/N	Sample of population from three fishing villages on the shore of Lake Victoria; Seweya (first visit)
Harada et al. 1999	Tanzania	14	3.79±9.87 (0.40-37.9)	Y/N	Seweya (first visit-outliers excluded)
Harada et al. 1999	Tanzania	20	28.9±98.2 (0.54-416)	Y/N	Seweya (second visit)
Harada et al. 1999	Tanzania	17	1.75±1.12 (0.54-3.9)	Y/N	Seweya (second visit-outliers excluded)
Harada et al. 1999	Tanzania	22	2.08±3.62 (0.42-17.4)	Y/N	Burgorola

Source	Location	N	Geometric Mean Hg ± SD (Range)[a]	Mining/Fish Consumers	Population Notes
Harada et al. 1999	Tanzania	26	1.00±0.60 (0.32-2.7)	Y/N	Hurseni
Harada et al. 1999	Tanzania	20	1.00±0.99 (0.29-5.1)	Y/N	Sample of population from a fishing village (Hombolo) facing a lake near Dodoma
Harada et al. 1999	Tanzania	19	59.1±129 (0.48-474)	Y/N	Sample of population of the city of Mwanza
Harada et al. 1999	Tanzania	15	3.44±6.98 (0.48-27.7)	Y/N	-Mwanza (outliers excluded)
Ikingura and Akagi 1996	Tanzania	22	0.947 (0.156-5.433)	Y/Y	Mugusu and Nungwe Bay area residents, Lake Victoria goldfields
Ikingura and Akagi 1996	Tanzania	2	34.212-214.188	Y/Y	Outliers from sampled residents; expected to be high due to extreme external contamination
Mohan et al. 2005	Suriname	39[b]	0.80 (0.10-15.40) (median)	Y/N	Mothers who delivered at Lands Hospitaal in Paramibo
Mohan et al. 2005	Suriname	39	1.60 (0.00-19.60) (median)	Y/N	Newborns born to mothers

[a]Data are geometric means, standard deviations, and ranges unless otherwise specified. [b]Females only.

Table S2. Studies that Reported Hair (µg/g) & Blood (µg/L) Mercury Concentrations Among Residents of ASGM Communities, Miners, and Environmentally Exposed Populations.

Source	Location	N	Media	Geometric Mean Hg ± SD (Range)[a]	Mining/Fish Consumers	Population Notes
Baeuml et al. 2011	Indonesia, Philippines, Tanzania, Zimbabwe & Mongolia	1174	Hair	5.71 (<LOD-792); 1.60 (median)	Y/Y	Participants ranged from individuals living in areas without gold mining to individuals with low, medium and high exposure to mercury from mining activities; Participants in Indonesia and the Philippines are more likely to be fish-consumers; participants in Tanzania, Zimbabwe and Mongolia consume less fish
Baeuml et al. 2011	Indonesia, Philippines, Tanzania, Zimbabwe & Mongolia	1121	Blood	11.1 (<LOD-429); 5.12 (median)	Y/Y	Participants ranged from individuals living in areas without gold mining to individuals with low, medium and high exposure to mercury from mining activities; Participants in Indonesia and the Philippines are more likely to be fish-consumers; participants in Tanzania, Zimbabwe and Mongolia consume less fish
Dolbec et al. 2000	Brazil	68	Hair	Males: 12.2±6.8; Females: 9.9±5.6	Y/Y	Tapajós riverbank village, 250 km downstream from gold mining
Dolbec et al. 2000	Brazil	68	Blood	Males: 40.7±23.32; Females: 33.0±27.6	Y/Y	Tapajós riverbank village, 250 km downstream from gold mining
Akagi et al. 1995	Brazil	27	Hair	THg: 24.6±17.8 MeHg: 24.1±17.8	Y/Y	Jacareacanga, Tapajós River basin residents; located downstream from Alta Floresta
Akagi et al. 1995	Brazil	19	Blood	THg: 90.4±71.5 MeHg: 90.0±76.6	Y/Y	Jacareacanga, Tapajós River basin residents; located downstream from Alta Floresta
Akagi et al. 1995	Brazil	14	Hair	THg: 37.4±17.1 MeHg: 36.4±17.1	Y/Y	Vila Sao Martins residents; isolated fishing village downstream from Alta Floresta
Akagi et al. 1995	Brazil	8	Blood	THg: 149.8±49.5 MeHg: 149.2±52.5	Y/Y	Vila Sao Martins residents; isolated fishing village downstream from Alta Floresta

Source	Location	N	Media	Geometric Mean Hg ± SD (Range)[a]	Mining/Fish Consumers	Population Notes
Akagi et al. 1995	Brazil	10	Hair	THg: 28.8±13.0<br>MeHg: 27.3±12.1	Y/Y	Vila Nova Sitio residents; isolated fishing village downstream from Alta Floresta
Akagi et al. 1995	Brazil	7	Blood	THg: 130.7±78.4<br>MeHg: 131.9±84.2	Y/Y	Vila Nova Sitio residents; isolated fishing village downstream from Alta Floresta
Akagi et al. 1995	Brazil	3	Hair	T-Hg: 4.1±1.3;<br>MeHg: 3.1±0.7	Y/N	Alta Floresta, one of the main gold trading centers in the Tapajos River basin; gold shop workers
Akagi et al. 1995	Brazil	25	Blood	THg: 12.2±8.2<br>MeHg: 9.0±6.7	Y/N	Alta Floresta, one of the main gold trading centers in the Tapajos River basin; gold shop workers
Nyland et al. 2011	Brazil	232	Hair	Median 14.1	Y/Y	Tapajós River residents
Nyland et al. 2011	Brazil	232	Blood	Mean 42.5; Median 53.5	Y/Y	Tapajós River residents
Counter et al. 2006	Ecuador	73	Hair	8.5±22.8 (1.0-135.0)	Y/N	Nambija and Portovelo gold mining areas; Andean children
Counter et al. 2006	Ecuador	73	Blood	5.1±2.4 (1.0-10.0)	Y/N	Nambija and Portovelo gold mining areas; Andean children
Bose-O'Reilly et al. 2008	Indonesia & Zimbabwe	31	Hair	1.23±0.81 (0.02-3.46)	N/N	Sulawesi, Indonesia & Kadoma, Zimbabwe; control group of children living in non-exposed areas
Bose-O'Reilly et al. 2008	Indonesia & Zimbabwe	31	Blood	2.95±2.21 (<0.20-7.94)	N/N	Sulawesi, Indonesia & Kadoma, Zimbabwe; control group of children living in non-exposed areas
Bose-O'Reilly et al. 2008	Indonesia & Zimbabwe	22	Hair	2.27±0.83 (0.42-4.16)	Y/N	Sulawesi, Indonesia & Kadoma, Zimbabwe children living in mercury-contaminated mining areas
Bose-O'Reilly et al. 2008	Indonesia & Zimbabwe	22	Blood	5.52±2.39 (1.28-12.40)	Y/N	Sulawesi, Indonesia & Kadoma, Zimbabwe children living in mercury-contaminated mining areas
Bose-O'Reilly et al. 2008	Indonesia & Zimbabwe	51	Hair	4.08±7.07 (0.45-52.96)	Y/N	Sulawesi, Indonesia & Kadoma, Zimbabwe children living in the mining area and working at least part-time with immediate contact to mercury
Bose-O'Reilly et al. 2008	Indonesia & Zimbabwe	51	Blood	12.40±14.66 (1.00-100.80)	Y/N	Sulawesi, Indonesia & Kadoma, Zimbabwe children living in the mining area and working at least part-time with immediate contact to mercury

Source	Location	N	Media	Geometric Mean Hg ± SD (Range)[a]	Mining/Fish Consumers	Population Notes
Bose-O'Reilly et al. 2010a	Indonesia	95	Hair	9.72 (0.58-239.04)	Y/N	Tatelu, Sulawesi residents (adults living in the mining area; adults working with mercury incl. mineral processors and amalgam-burners)
Bose-O'Reilly et al. 2010a	Indonesia	95	Blood	21.38 (3.42-186.00)	Y/N	Tatelu, Sulawesi residents (adults living in the mining area; adults working with mercury incl. mineral processors and amalgam-burners)
Bose-O'Reilly et al. 2010a	Indonesia	21	Hair	1.64 (0.83-3.72)	N/N	Air Mandidi, Sulawesi; control group
Bose-O'Reilly et al. 2010a	Indonesia	21	Blood	4.92 (2.36-10.12)	N/N	Air Mandidi, Sulawesi; control group
Bose-O'Reilly et al. 2010a	Indonesia	165	Hair	17.75 (0.33-792.45)	Y/N	Galangan, Kalimantan residents (adults living in the mining area; adults working with mercury incl. mineral processors and amalgam-burners)
Bose-O'Reilly et al. 2010a	Indonesia	165	Blood	25.12 (1.45-429.00)	Y/N	Galangan, Kalimantan residents (adults living in the mining area; adults working with mercury incl. mineral processors and amalgam-burners)
Bose-O'Reilly et al. 2010b	Tanzania	31	Hair	0.36 (0.08-0.68 (95th percentile 0.65))	N/N	Katoro residents; control group
Bose-O'Reilly et al. 2010b	Tanzania	31	Blood	1.05 (0.22-2.29)	N/N	Katoro residents; control group
Bose-O'Reilly et al. 2010b	Tanzania	52	Hair	0.85 (0.12-15.75)	Y/N	Living in exposed area but no work contact with mercury
Bose-O'Reilly et al. 2010b	Tanzania	52	Blood	1.51 (0.45-5.84)	Y/N	Living in exposed area but no work contact with mercury
Bose-O'Reilly et al. 2010b	Tanzania	34	Hair	0.84 (0.24-4.07)	Y/N	Working with mercury but not smelting
Bose-O'Reilly et al. 2010b	Tanzania	34	Blood	1.98 (0.71-6.06)	Y/N	Working with mercury but not smelting
Bose-O'Reilly et al. 2010b	Tanzania	104	Hair	2.69 (0.16-48.74)	Y/N	Working with mercury including smelting

Source	Location	N	Media	Geometric Mean Hg ± SD (Range)[a]	Mining/Fish Consumers	Population Notes
Bose-O'Reilly et al. 2010b	Tanzania	104	Blood	4.62 (0.73-33.30)	Y/N	Working with mercury including smelting
Steckling et al. 2011	Mongolia	92[b]	Hair	0.24 (0.10-1.61)	Y/N	Residents with low exposure (environmental, no occupational)
Steckling et al. 2011	Mongolia	92[b]	Blood	0.32 (<LOD -7.60)	Y/N	Residents with low exposure (environmental, no occupational)
Steckling et al. 2011	Mongolia	64[b]	Hair	0.34 (0.10-2.71)	Y/N	Residents with active occupational exposure
Steckling et al. 2011	Mongolia	64[b]	Blood	0.55 (<LOD -9.60)	Y/N	Residents with active occupational exposure
Steckling et al. 2011	Mongolia	35[b]	Hair	0.13 (0.10-0.62)	N/N	Kuhshaat sum residents; control group
Steckling et al. 2011	Mongolia	42[b]	Blood	0.3 (<LOD -3.60)	N/N	Kuhshaat sum residents; control group
Akagi et al. 2000	Philippines	162	Hair	THg: 0.99±1.6 (0.33-20.39); MeHg: 0.80±1.45 (0.19-18.47) (µg/g)	Y/N	Gold processing and refining area; elementary school children
Akagi et al. 2000	Philippines	162	Blood	THg: 0.0038±0.0046 (0.00076-0.057); MeHg: 0.006±0.0083 (0.0014-0.047) (µg/g)	Y/N	Gold processing and refining area; elementary school children
Drasch et al. 2001	Philippines	316	Hair	4.14 (AM), 2.72 (median), (0.03-37.76)	Y/Y	Gold mining workers, inhabitants of a gold mining area, individuals living downstream and controls
Drasch et al. 2001	Philippines	323	Blood	11.48 (AM), 8.2 (median), (<0.25-107.60)	Y/Y	Gold mining workers, inhabitants of a gold mining area, individuals living downstream and controls
Ramirez et al. 2003	Philippines	48	Hair	1.28±0.30	Y/Y	Tagum (small-scale gold mining area) infants
Ramirez et al. 2003	Philippines	48	Blood	2.60±0.27	Y/Y	Tagum (small-scale gold mining area) infants
Ramirez et al. 2003	Philippines	88	Hair	0.66±0.05	N/Y	Saranggani, no known sources of industrial, chemical, or pesticide pollution; children

Source	Location	N	Media	Geometric Mean Hg ± SD (Range)[a]	Mining/Fish Consumers	Population Notes
Ramirez et al. 2003	Philippines	88	Blood	3.25±0.22	N/Y	Saranggani, no known sources of industrial, chemical, or pesticide pollution; children
Cortes-Maramba et al. 2006	Philippines	18	Hair	0.008±0.016 (0.0018-0.069)	Y/N	Sibutad (small-scale gold mining community); individuals directly exposed by blow torching or amalgam squeezing
Cortes-Maramba et al. 2006	Philippines	18	Blood	14.89±6.44 (5.82-29.48)	Y/N	Sibutad (small-scale gold mining community); individuals directly exposed by blow torching or amalgam squeezing
Cortes-Maramba et al. 2006	Philippines	20	Hair	0.0025±0.001 (0.001-0.005)	Y/N	Indirect exposure to mercury
Cortes-Maramba et al. 2006	Philippines	20	Blood	9.26±4.45 (3.38-20.57)	Y/N	Indirect exposure to mercury
Adimado & Baah 2002	Ghana	50	Hair	1.61±1.33 (0.15-5.86)	Y/Y	Anwiaso (upstream), Ankobra river basin residents
Adimado & Baah 2002	Ghana	50	Blood	102.0±55.8 (30.2-218)	Y/Y	Anwiaso (upstream), Ankobra river basin residents
Adimado & Baah 2002	Ghana	50	Hair	0.62±0.41 (0.32-2.19)	Y/Y	Sahuma (downstream); Ankobra river basin residents
Adimado & Baah 2002	Ghana	50	Blood	13.4±15.3 (2.1-68.1)	Y/Y	Sahuma (downstream); Ankobra river basin residents
Adimado & Baah 2002	Ghana	51	Hair	4.27±6.26 (0.06-28.3)	Y/Y	Tanoso (upstream), Tano river basin residents
Adimado & Baah 2002	Ghana	51	Blood	16.5±10.7 (2.1-57.2)	Y/Y	Tanoso (upstream), Tano river basin residents
Adimado & Baah 2002	Ghana	66	Hair	1.21±0.65 (0.07-3.19)	Y/Y	Elubo (downstream), Tano river basin residents
Adimado & Baah 2002	Ghana	66	Blood	39.5±16.2 (1.8-70.4)	Y/Y	Elubo (downstream), Tano river basin residents

Abbreviation: AM, arithmetic mean

[a]Data are geometric means, standard deviations, and ranges unless otherwise specified. [b]Females only.

Table S3. Studies that Reported Blood Mercury Concentrations (µg/L) Among Residents of ASGM Communities, Miners, and Environmentally Exposed Populations.

Source	Location	N	Geometric Mean Hg ± SD (Range)[a]	Mining/Fish Consumers	Population Notes
Yard et al. 2012	Peru	103	Males: 2.60;<br>Females: 1.91	Y/Y	Residents of Huaypetu, an artisanal mining town
Ramirez et al. 2000	Philippines	12	53.3±37.49 (20-130)	Y/Y	Tagum (small-scale gold mining area); fetal cord blood
Ramirez et al. 2000	Philippines	5	24±5.47 (20-30)	Y/Y	mothers
de Kom et al. 1998	Suriname	25	18.1±11.0	Y/N	Sillakreek mining area workers
de Kom et al. 1998	Suriname	16	26.8±14.6	Y/N	Control group; residents of Djumu
Oosthuizen et al. 2010	South Africa	20	2.35 (median) (<0.5-24.0)	Y/Y	Community in Mpumalanga; in a gold-mining area
Harari et al. 2012	Ecuador	200	30.1 (4.4-89)	Y/N	Gold miners (product gold amalgam and intermittently burn gold amalgam)
Harari et al. 2012	Ecuador	37	5.3 (0.7-100)	Y/N	Gold merchants (burn amalgam daily)
Harari et al. 2012	Ecuador	72	5.0 (2.0-13)	N/N	Referents (workers in a factory refining gold by a non-mercury method or workers with other jobs not occupationally exposed to mercury)

[a]Data are geometric means, standard deviations, and ranges unless otherwise specified.

Table S4. Studies that Reported Urinary Mercury Concentrations (µg/g-Cr unless otherwise specified) Among Residents of ASGM Communities and Miners.

Source	Location	N	Geometric Mean Hg ± SD (Range)[a]	Mining/Fish Consumers	Population Notes
Baeuml et al. 2011	Indonesia, Philippines, Tanzania, Zimbabwe & Mongolia	1178	18.6 (<LOD-1697)<br>3.08 (median)	Y/Y	Participants ranged from individuals living in areas without gold mining to individuals with low, medium and high exposure to mercury from mining activities; Participants in Indonesia and the Philippines are more likely to be fish-consumers; participants in Tanzania, Zimbabwe and Mongolia consume less fish
Yard et al. 2012	Peru	102	M 5.74<br>F 5.19	Y/Y	Residents of Huaypetu, an artisanal mining town (total)
Yard et al. 2012	Peru	30	7.36	Y/Y	Individuals involved in mixing mercury with gold ore
Yard et al. 2012	Peru	72	4.84	Y/Y	Individuals not involved in mixing mercury with gold ore
Yard et al. 2012	Peru	30	8.81	Y/Y	Individuals who heat gold-mercury amalgams
Yard et al. 2012	Peru	72	4.49	Y/Y	Individuals who do not heat gold-mercury amalgams
Harari et al. 2012	Ecuador	200	3.3 (0.3-170)	Y/N	Gold miners (product gold amalgam and intermittently burn gold amalgam)
Harari et al. 2012	Ecuador	37	36.9 (3.2-420)	Y/N	Gold merchants (burn amalgam daily)
Harari et al. 2012	Ecuador	72	1.6 (0.2-13)	N/N	Referents (workers in a factory refining gold by a non-mercury method or workers with other jobs not occupationally exposed to mercury)
Tomicic et al. 2011	Burkina Faso	52	299.1 (AM), 174.3 (GM)	Y/N	Unrefined gold dealers
Tomicic et al. 2011	Burkina Faso	33	73.3 (AM), 37.7 (GM)	Y/N	Workers involved with ore washing and amalgamation
Tomicic et al. 2011	Burkina Faso	8	14.6 (AM), 12.4 (GM)	Y/N	Other workers involved with crushing, grinding and washing
de Kom et al. 1998	Suriname	28	27.5±21.1	Y/N	Sillakreek mining area workers
de Kom et al. 1998	Suriname	17	5.2±2.9	Y/N	Control group; residents of Djumu

Source	Location	N	Geometric Mean Hg ± SD (Range)[a]	Mining/Fish Consumers	Population Notes
Steckling et al. 2011	Mongolia	85	7.83 (0.16-78.7)	Y/N	Residents with low exposure (environmental, no occupational)
Steckling et al. 2011	Mongolia	58	18.81 (0.15-311)	Y/N	Residents with medium to high exposure (active occupational exposure)
Kwaansa-Ansah et al. 2010	Ghana	40	1.23±0.86 (0.32-3.62) µg/L	Y/Y	Dunkwa-On-Offin; miners
Kwaansa-Ansah et al. 2010	Ghana	54	0.69±0.39 (0.075-2.31) µg/L	Y/Y	Sample of farmers
Paruchuri et al. 2010	Ghana	120	17±77.3 (0.2-708.0) µg/L	Y/N	Talensi-Nabdam district, Upper East region; miners and residents
Oosthuzien et al. 2010	South Africa	28	6.2 (median) (0.5-63.5)	Y/Y	Community in Mpumalanga; in a gold-mining area
Bose-O'Reilly et al. 2008	Indonesia & Zimbabwe	50	0.35±0.26	N/N	Sulawesi, Indonesia & Kadoma, Zimbabwe; control group of children living in non-exposed areas
Bose-O'Reilly et al. 2008	Indonesia & Zimbabwe	36	9.16±11.32	Y/N	Sulawesi, Indonesia & Kadoma, Zimbabwe children living in mercury-contaminated mining areas
Bose-O'Reilly et al. 2008 (cont.)	Indonesia & Zimbabwe	80	36.50±93.06	Y/N	Sulawesi, Indonesia & Kadoma, Zimbabwe children living in the mining area and working at least part-time with immediate contact to mercury (i.e., panning or amalgam burning)
Umbangtalad et al. 2007	Thailand	n/a	5.34±0.10	N/N	Individuals residing in non-gold mining areas with no exposure; control group
Umbangtalad et al. 2007	Thailand	17	32.02±0.20	Y/N	Phanom Pha; miners who worked in the amalgamation process
Umbangtalad et al. 2007	Thailand	62	20.04±0.49	Y/N	Phanom Pha; miners who worked in the ore preparation area
Umbangtalad et al. 2007	Thailand	40	15.82±0.04	Y/N	Phanom Pha; elementary school students – involved directly or indirectly in gold mining activities
Umbangtalad et al. 2007	Thailand	19	9.95±0.07	Y/N	Phanom Pha; elementary school students not involved or associated with mining

Source	Location	N	Geometric Mean Hg ± SD (Range)[a]	Mining/Fish Consumers	Population Notes
Counter et al. 2005	Ecuador	80	10.9±21.7 (1-166) µg/L	Y/N	Nambija; Andean children of Saraguro and Metizo gold miners
Counter et al. 2006	Ecuador	73	13.3±25.9 (1-166) µg/L	Y/N	Nambija and Portovelo gold mining areas; Andean children
Drake et al. 2001	Venezuela	8	11.9±16.1 (2.5-49.9)	Y/N	Large mining company employees
Drake et al. 2001	Venezuela	21	148±240 (2.5-912)	Y/N	Informal or "self-employed" miners
Drake et al. 2001	Venezuela	4	39.1±58.9 (2.5-127)	Y/N	Jewelers
Bose-O'Reilly et al. 2010b	Tanzania	31	0.24 (0.04-0.92)	N/N	Control group
Bose-O'Reilly et al. 2010b	Tanzania	52	0.77 (0.05-11.62)	Y/N	Living in exposed area but no work contact with mercury
Bose-O'Reilly et al. 2010b	Tanzania	34	1.21 (0.11-7.00)	Y/N	Working with mercury but no smelting
Bose-O'Reilly et al. 2010b	Tanzania	104	6.15 (0.12-36.77)	Y/N	Working with mercury including smelting
Bose-O'Reilly et al. 2010a	Indonesia	21	0.43 (0.09-1.35)	N/N	Sulawesi; control group
Bose-O'Reilly et al. 2010a	Indonesia	66	10.44 (0.46-355.30)	Y/N	Kalimantan; only living in exposed area
Bose-O'Reilly et al. 2010a	Indonesia	18	2.70 (0.70-9.84)	Y/N	Sulawesi; only living in exposed area
Bose-O'Reilly et al. 2010a	Indonesia	30	15.65 (0.47-261.94)	Y/N	Kalimantan; worker (panning)
Bose-O'Reilly et al. 2010a	Indonesia	17	5.58 (0.83-22.95)	Y/N	Sulawesi; worker (panning)
Bose-O'Reilly et al. 2010a	Indonesia	69	69.35 (0.72-1697.39)	Y/N	Kalimantan; worker (smelting)
Bose-O'Reilly et al. 2010a	Indonesia	60	31.89 (0.48-232.82)	Y/N	Sulawesi; worker (smelting)

Source	Location	N	Geometric Mean Hg ± SD (Range)[a]	Mining/Fish Consumers	Population Notes
Drasch et al. 2001	Philippines	42	1.4 (median); 9.3 (maximum)	N/N	Control group; residents of Davao
Drasch et al. 2001	Philippines	100	1.4 (median); 5.2 (maximum)	N/Y	Individuals living in the Monkayo area, downstream of Mt. Diwata
Drasch et al. 2001	Philippines	63	4.1 (median); 76.4 (maximum)	N/Y	Individuals living in Diwalwal, but no occupational exposure
Drasch et al. 2001	Philippines	102	11.0 (median); 294.2 (maximum)	Y/Y	Individuals living in Diwalwal with occupational exposure working with gold

Abbreviations: AM, arithmetic mean; GM, geometric mean

[a]Data are geometric means, standard deviations, and ranges unless otherwise specified.

References

Adimado AA, Baah DA. 2002. Mercury in human blood, urine, hair, nail, and fish from the Ankobra and Tano River Basins in southwestern Ghana. Bull Environ Contam Toxicol 68(3):339-346.

Akagi H, Malm O, Kinjo Y, Harada M, Branches FJP, Pfeiffer WC, et al. 1995. Mercury pollution in the Amazon, Brazil. Sci Total Environ 175(2):85-95.

Ashe K. 2012. Elevated mercury concentrations in humans of Madre de Dios, Peru. PLoS One 7(3):e33305; doi: 10.1371/journal.pone.0033305 [Online 16 March 2012].

Baeuml, J, Bose-O'Reilly S, Matteucci Gothe R, Lettmeier B, Roider G, Drasch G, Siebert U. 2011. Human biomonitoring data from mercury exposed miners in six artisanal small-scale gold mining areas in Asia and Africa. Minerals 1(1):122-143.

Barbieri FL, Cournil A, Gardon J. 2009. Mercury exposure in a high fish eating Bolivian Amazonian population with intense small-scale gold-mining activities. Int J Environ Health Res 19(4):267-277.

Barbosa AC, Garcia AM, de Souza JR. 1997. Mercury contamination in hair of riverine populations of Apiacás Reserve in the Brazilian Amazon. Water Air Soil Pollut 97:1-8.

Barbosa AC, Silva SRL, Dórea JG. 1998. Concentration of Mercury in Hair of Indigenous Mothers and Infants from the Amazon Basin. Arch Environ Contam Toxicol 34(1):100–105.

Bose-O'Reilly S, Lettmeier B, Gothe RM, Beinhoff C, Siebert U, Drasch G. 2008. Mercury as a serious health hazard for children in gold mining areas. Environ Res 107:89-97.

Bose-O'Reilly S, Drasch G, Beinhoff C, Rodrigues-Filho S, Roider G, Lettmeier B, et al. 2010a. Health assessment of artisanal gold miners in Indonesia. Sci Total Environ 408(4):713-25.

Bose-O'Reilly S, Drasch G, Beinhoff C, Tesha A, Drasch K, Roider G, et al. 2010b. Health assessment of artisanal gold miners in Tanzania. Sci Total Environ 408(4):796-805.

Cordier S, Grasmick C, Paquier-Passelaique M, Manderau L, Weber JP, Jouan M. 1998. Mercury exposure in French Guiana: levels and determinants. Arch Environ Health 53(4):299-303.

Cordier S, Garel M, Mandereau L, Morcel H, Doineau P, Gosme-Seguret S, et al. 2002. Neurodevelopmental investigations among methymercury-exposed children in French Guiana. Environ Res Section A 89:1-11.

Cortes-Maramba N, Reyes JP, Francisco-Rivera AT, Akagi H, Sunio R, Panganiban LC. 2006. Health and environmental assessment of mercury exposure in a gold mining community in Western Mindanao, Philippines. J Environ Manage 81(2):126-134.

Counter SA, Buchanan LH, Ortega F. 2005. Mercury levels in urine and hair of children in an Andean gold-mining settlement. Int J Occup Environ Health 11(2):132-137.

Counter SA, Buchanan LH, Ortega F. 2006. Neurocognitive screening of mercury-exposed children of Andean gold miners. Int J Occup Environ Health 12:209-214.

Crompton P, Ventura AM, de Souza JM, Santos E, Strickland GT, Silbergeld E. 2002. Assessment of mercury exposure and malaria in a Brazilian Amazon riverine community. Environ Res 90(2):69-75.

de Kom JF, van der Voet GB, de Wolff FA. 1998. Mercury exposure of maroon workers in small scale gold mining in Suriname. Environ Res 77(2):91-97.

Dolbec J, Mergler D, Sousa Passos CJ, Sousa de Morais S, Lebel J. 2000. Methylmercury exposure affects motor performance of a riverine population of the Tapajós river, Brazilian Amazon. Int Arch Occup Environ Health 73:195-203.

Donkor AK, Bonzongo JC, Nartey VK, Adotey DK. 2006. Mercury in different environmental compartments of the Pra River Basin, Ghana. Sci Total Environ 368(1):164-176.

Dórea JG, Barbosa AC, Ferrari I, de Souza JR. 2005a. Fish consumption (hair mercury) and nutritional status of Amazonian Amer-Indian children. Am J Hum Biol 17(4):507-514.

Dórea JG, de Souza JR, Rodrigues P, Ferrari I, Barbosa AC. 2005b. Hair mercury (signature of fish consumption) and cardiovascular risk in Munduruku and Kayabi Indians of Amazonia. Environ Res 97(2):209-219.

Dórea JG, Marques RC, Isejima C. 2012. Neurodevelopment of Amazonian infants: antenatal and postnatal exposure to methyl- and ethylmercury. J Biomed Biotechnol, doi; 10.1155/2012/132876 [Online 26 April 2012].

Drake PL, Rojas M, Reh CM, Mueller CA, Jenkins FM. 2001. Occupational exposure to airborne mercury during gold mining operations near El Callao, Venezuela. Int Arch Occup Environ Health 74:206-212.

Drasch G, Böse-O'Reilly S, Beinhoff C, Roider G, Maydl S. 2001. The Mt. Diwata study on the Philippines 1999 – assessment mercury intoxication of the population by small scale gold mining. Sci Total Environ 267(1-3):151-168.

Fréry N, Maury-Brachet R, Maillot E, Deheeger M, de Mérona B, Boudou A. 2001. Gold-mining activities and mercury contamination of native Amerindian communities in French Guiana: key role of fish in dietary uptake. Environ Health Perspect 109(5):449-456.

Fujimura M, Matsuyama A, Harvard JP, Bourdineaud JP, Nakamura K. 2012. Mercury contamination in humans in Upper Maroni, French Guiana between 2004 and 2009. Bull Environ Contam Toxicol 88(2):135-139.

Grandjean P, White RF, Nielsen A, Cleary D, Oliveira Santos EC. 1999. Methylmercury neurotoxicity in Amazonian children downstream from gold mining. Environ Health Perspect 107(7):587-591.

Guimarães JRD, Fostier AH, Forti MC, Melfi JA, Kehrig H, Mauro JBN, et al. 1999. Mercury in human and environmental samples from two lakes in Amapá, Brazilian Amazon. Ambio 28(4):296-301.

Hacon S, Yokoo E, Valente J, Campos RC, da Silva VA, de Menezes AC et al. 2000. Exposure to mercury in pregnant women from Alta Floresta-Amazon basin, Brazil. Environ Res 84(3):204-210.

Harada M, Nakachi S, Cheu T, et al. 1999. Monitoring of mercury pollution in Tanzania: relation between head hair mercury and health. Sci Total Environ 227(2-3):249-256.

Harada M, Nakanishi J, Yasoda E, Pinheiro MC, Oikawa T, de Assis Guimarães G et al. 2001. Mercury pollution in the Tapajos River basin, Amazon: mercury level of head hair and health effects. Environ Int 27(4):285-290.

Harari R, Harari F, Gerhardsson L, Lundh T, Skerfving S, Strömberg, Broberg K. 2012. Exposure and toxic effects of elemental mercury in gold-mining activities in Ecuador. Toxicol Lett 213:75-82.

Ikingura JR, Akagi H. 1996. Monitoring of fish and human exposure to mercury due to gold mining in the Lake Victoria goldfields, Tanzania. Sci Total Environ 191(1-2):59-68.

Kwaansa-Ansah EE, Basu N, Nriagu JO. 2010. Environmental and occupational exposures to mercury among indigenous people in Dunkwa-On-Offin, a small scale gold mining area in the South-West of Ghana. Bull Environ Contam Toxicol 85(5):476-480.

Lasut MT, Yasuda Y, Edinger EN, Pangemanan JM. 2010. Distribution and accumulation of mercury derived from gold mining in marine environment and its impact on residents of Buyat Bay, North Sulawesi, Indonesia. Water Air Soil Pollut 208:153-164.

Lebel J, Roulet M, Mergler D, Lucotte M, Larribe F. 1997. Fish diet and mercury exposure in a riparian Amazonian population. Water Air Soil Pollut 97:31-44.

Leino T, Lodenius M. 1995. Human hair mercury levels in Tucuruí, State of Pará, Brazil. Sci Total Environ 175(2):119-125.

Malm O, Branches FJP, Akagi H, Castro MB, Pfeiffer WC, Harada M, et al. 1995. Mercury and methylmercury in fish and human hair from the Tapajós river basin, Brazil. Sci Total Environ 175:141-150.

Malm O, Dórea JG, Barbosa AC, Pinto FN, Weihe P. 2010. Sequential hair mercury in mothers and children from a traditional riverine population of the Rio Tapajós, Amazonia: Seasonal changes. Environ Res 110(7):705-709.

Maurice-Bourgoin L, Quiroga I, Chincheros J, Courau P. 2000. Mercury distribution in waters and fishes of the upper Madeira rivers and mercury exposure in riparian Amazonian populations. Sci Total Environ 260(1-3):73-86.

Mohan S, Tiller M, van der Voet G, Kanhai H. 2005. Mercury exposure of mothers and newborns in Surinam: a pilot study. Clin Toxicol (Phila) 43(2):101-104.

Monrrroy SX, Lopez RW, Roulet M, Benefice E. 2008. Lifestyle and mercury contamination of Amerindian populations along the Beni river (lowland Bolivia). J Environ Health 71(4):44-50.

Murphy TP, Irvine KN, Sampson M, Guo J, Parr T. 2008. Mercury contamination along the Mekong River, Cambodia. Asian Journal of Water, Environment and Pollution 6(1):1-9.

Nyland JF, Fillion M, Barbosa F Jr., Shirley DL, Chine C, Lemire M, et al. 2011. Biomarkers of methylmercury exposure immunotoxicity among fish consumers in Amazonian Brazil. Environ Health Perspect 119:1733-1738.

Olivero-Verbel J, Caballero-Gallardo K, Marrugo Negrete J. 2011. Relationship between localization of gold mining areas and hair mercury levels in people from Bolivar, north of Colombia. Biol Trace Elem Res 144(1-3):118-132.

Oosthuizen MA, John J, Somerset V. 2010. Mercury exposure in a low-income community in South Africa. S Afr Med J 100(6):366-371.

Paruchuri Y, Siuniak A, Johnson N, Levin E, Mitchell K, Goodrich JM, et al. 2010. Occupational and environmental mercury exposure among small-scale gold miners in the Talensi–Nabdam District of Ghana's Upper East region. Sci Total Environ 408(24):6079-6085.

Peplow D, Augustine S. 2012. Community-led assessment of risk from exposure to mercury by native Amerindian Wayana in Southeast Suriname. J Environ Public Health; doi:10.1155/2012/674596 [Online 15 November 2011].

Pinheiro MC, Müller RC, Sarkis JE, et al. 2005. Mercury and selenium concentrations in hair samples of women in fertile age from Amazon riverside communities. Sci Total Environ 349(1-3):284-288.

Pinheiro MC, Oikawa T, Vieira JL, et al. 2006. Comparative study of human exposure to mercury in riverside communities in the Amazon region. Braz J Med Biol Res 39(3):411-414.

Pinheiro MCN, Crespo-López ME, Vieira JLF, Oikawa T, Guimarães GA, Araújo CC, et al. 2007. Mercury pollution and childhood in Amazon riverside villages. Environ Int 33(1):56–61.

Ramirez GB, Cruz MC, Pagulayan O, Ostrea E, Dalisay C. 2000. The Tagum study I: Analysis and clinical correlates of mercury in maternal and cord blood, breast milk, meconium, and infants' hair. Pediatrics 106(4):774-781.

Ramirez GB, Pagulayan O, Akagi J, Rivera AF, Eng C, Lee LV, et al. 2003. Tagum study II: Follow-up study at two years of age after prenatal exposure to mercury. Pediatrics 111(3):e289-e295.

Saeki K, Fujimoto M, Kolinjim D, Tatsukawa R. 1996. Mercury concentrations in hair from populations in Wau-Buolo area, Papua New Guinea. Arch Environ Contam Toxicol 30(3):412-417.

Sakamoto, M. 2004. Mercury Pollution, Buyat & Totok Bay, North Sulawesi, Indonesia, Final Report: Prepared by Dr. Sakamoto of Institute for Minamata Disease, Min. of Environ., Japan, for World Health Organization, Sept. 8, 2004, 25 pg.

Santos EC, de Jesus IM, Câmara Vde M, Brabo E, Loureiro EC, Mascarenhas A, et al. 2002. Mercury exposure in Munduruku Indians from the community of Sai Cinza, State of Pará, Brazil. Environ Res 90(2):98-103.

Steckling N, Bose-O'Reilly S, Gradel C, Gutschmidt K, Shinee E, Altangerel E, et al. 2011. Mercury exposure in female artisanal small-scale gold miners (ASGM) in Mongolia: An analysis of human biomonitoring (HBM) data from 2008. Sci Total Environ 409(5):994-1000.

Tomicic C, Vernez D, Belem T, Berode M. 2011. Human mercury exposure associated with small-scale gold mining in Burkina Faso. Int Arch Occup Environ Health 84(5):539-546.

Umbangtalad S, Parkpian P, Visvanathan C, Delane RD, Jugsujinda A. 2007. Assessment of Hg contamination and exposure to miners and schoolchildren at a small-scale gold mining and recovery operation in Thailand. J Environ Sci Health A Tox Hazard Subst Environ Eng 42(14):2071-2079.

van Straaten P. 2000. Human exposure to mercury due to small scale gold mining in northern Tanzania. Sci Total Environ 259(1-3):45-53.

Williams TM, Apostol AN, Miranda CR. 2000. Assessment by hair analysis of mercury exposure among mining impacted communities of Mindanao and Palawan, The Philippines. Env Geochem Health 22(1):19-31.

Yard EE, Horton J, Schier JG, Caldwell K, Sanchez C, Lewis L, Gastañaga C. 2012. Mercury exposure among artisanal gold miners in Madre de Dios, Peru: A cross-sectional study. J Med Toxicol 8(4):441-448.
